# Prevalence of Steatosis Hepatis in the* Eurotransplant* Region: Impact on Graft Acceptance Rates

**DOI:** 10.1155/2018/6094936

**Published:** 2018-11-01

**Authors:** Simon Moosburner, Joseph M. G. V. Gassner, Maximilian Nösser, Julian Pohl, David Wyrwal, Felix Claussen, Paul V. Ritschl, Duska Dragun, Johann Pratschke, Igor M. Sauer, Nathanael Raschzok

**Affiliations:** ^1^Department of Surgery, Campus Charité Mitte ∣ Campus Virchow-Klinikum, Experimental Surgery and Regenerative Medicine, Charité – Universitätsmedizin Berlin 13353, Germany; ^2^BIH Charité Clinician Scientist Program, Berlin Institute of Health (BIH), Berlin 10178, Germany; ^3^Berlin Institute of Health and Department of Nephrology and Critical Care Medicine, Charité Universitätsmedizin, Berlin 13353, Germany

## Abstract

Due to the shortage of liver allografts and the rising prevalence of fatty liver disease in the general population, steatotic liver grafts are considered for transplantation. This condition is an important risk factor for the outcome after transplantation. We here analyze the characteristics of the donor pool offered to the* Charité – Universitätsmedizin Berlin* from 2010 to 2016 with respect to liver allograft nonacceptance and steatosis hepatis. Of the 2653 organs offered to our center, 19.9% (n=527) were accepted for transplantation, 58.8% (n=1561) were allocated to other centers, and 21.3% (n = 565) were eventually discarded from transplantation. In parallel to an increase of the incidence of steatosis hepatis in the donor pool from 20% in 2010 to 30% in 2016, the acceptance rates for steatotic organs increased in our center from 22.3% to 51.5% in 2016 (p < 0.001), with the majority (86.9%; p > 0.001) having less than 30% macrovesicular steatosis hepatis. However, by 2016, the number of canceled transplantations due to higher grades of steatosis hepatis had significantly increased from 14.7% (n = 15) to 63.6% (42; p < 0.001). The rising prevalence of steatosis hepatis in the donor pool has led to higher acceptance rates of steatotic allografts. Nonetheless, steatosis hepatis remains a predominant phenomenon in discarded organs necessitating future concepts such as organ reconditioning to increase graft utilization.

## 1. Introduction

Orthotopic liver transplantation (OLT), which is the only curative therapy option in patients with end-stage liver disease, is increasingly limited by the discrepancy between organ demand and availability [1, 2]. Donation after cardiac death, split-liver transplantation, living donor liver transplantation, and transplantation of grafts from extended criteria donors have been developed to expand the donor pool [3, 4]. In spite of these developments, and due to the increase in donor age and stagnation of donations, the number of patients on the waiting list constantly exceeds the organ supply [5]. While the number of liver transplantations decreased, more restrictive listing policies have led to sicker patients on the waiting list, with high rates of mortality and impaired outcome after liver transplantation [6–8].

Steatosis hepatis, also known as fatty liver disease, is considered an important risk factor for graft dysfunction after liver transplantation, and more than 50% of grafts with histologically confirmed moderate or severe macrosteatosis are usually not used for transplantation [9]. Nonalcoholic fatty liver disease, which is the hepatic manifestation of the metabolic syndrome, is already the second most common cause for liver transplantation in the USA and currently the only increasing etiology with increasing incidence [10–13]. With the rising prevalence of steatosis hepatis in potential donors, graft utilization is expected to fall from 78% to 44% by 2030 [10]. However, data on the current nonacceptance rate of liver grafts due to steatosis hepatis in the* Eurotransplant* region are not well documented in the literature. Based on large retrospective database analyses, transplantation of liver grafts with macrovesicular steatosis > 30% is only recommended from donors with less overall risk factors [14, 15]. Even though macrovesicular steatosis is a recognized risk factor for primary nonfunction and early allograft dysfunction (EAD) [14–21], the extent of the postoperative impairment remains disputed. It is generally accepted that severe macrovesicular steatosis ≥ 60% leads to higher rates of primary nonfunction and EAD, and to reduced 1- and 3-year recipient and graft survival [16, 22, 23], while mild steatotic organs seem to be safe to transplant [14, 15, 24].

Germany in particular has seen a drastic 30% decline in organ donation, from 1200 donors in 2011 to only 857 donors in 2016. This aggravates the need to offer grafts from extended criteria donors to meet the demand for liver allografts. The question arises if expanding the donor pool with such donors has actually yielded higher rates of transplantations or just higher rates of notaccepted livers. To address this question and to update the knowledge concerning liver graft utilization and reasons for nonacceptance in the* Eurotransplant* region [25], we here analyzed all grafts offered to our high-volume center from 2010 to 2016 with regard to allocation, i.e., acceptance, nonacceptance, or discarded organs, with a special focus on grafts with steatosis hepatis.

## 2. Materials and Methods

### 2.1. Study Site and Ethical Board Approval

This single center retrospective data analysis was performed in the Department of Surgery, Campus Charité Mitte ∣ Campus Virchow-Klinikum of the Charité – Universitätsmedizin Berlin (Berlin, Germany). The study protocol was approved by the local ethics committee (Ethics committee of the Charité, EA2/010/17).

### 2.2. Organ Offers

Data for all livers from 2010 to 2016 offered by* Eurotransplant* to the Charité – Universitätsmedizin Berlin was requested from* Eurotransplant* and analyzed. All donors included in the analysis were from brain death donors (DBD). Donor data included in analysis were donor age, body mass index (BMI), hepatitis B (HBV) status, hepatitis C (HCV) status, aspartate-aminotransferase (AST), alanine-aminotransferase (ALT), gamma-glutamyl transferase (GGT), international normalized ratio (INR), c-reactive protein (CRP), creatinine, sodium, history of diabetes mellitus, or smoking, cardiopulmonary resuscitation, cause of death, duration of intensive care unit (ICU) stay, signs for steatosis hepatis in ultrasonography, steatosis hepatis in histopathology report, and the allocation phase, i.e., whether the offered donor liver was procured or transplanted at all.

### 2.3. Organ Acceptance

All liver offers made to the Charité – Universitätsmedizin Berlin are recorded and in case of nonacceptance the reason is remarked. Clinic records were screened from 2010 to 2016. Reason of nonacceptance was categorized into “donor medical,” “weight/size,” “recipient medical,” “logistics,” or “other reasons.” “Donor medical” was further subclassified into “age,” “biochemical parameters,” “cardiopulmonary resuscitation,” “steatosis hepatis,” “infection,” “malignancy,” “substance abuse,” “ICU stay,” or “other reasons.” Liver allografts were accepted on a case-by-case basis for each individual patient, considering donor age, weight, and size relative to recipient age, as well as the virologic status of the donor (especially HCV and human immunodeficiency virus). Additionally, the expected cold ischemia time (CIT) and the presence of steatosis hepatis influenced the acceptance decision.

### 2.4. Classification of Steatosis Hepatis

Steatosis hepatis is classified into two groups: (I) any description of steatosis hepatis, i.e., ultrasound or histopathological report; (II) cases with histopathological confirmation. The histopathological confirmed cases were further graded by the degree of macrovesicular steatosis hepatis as previously reported by Chu et al. and Briceño et al. [24, 26]. Macrovesicular steatosis < 5% was classified as “no steatosis,” followed by < 30% as “mild steatosis,” ≥ 30% as “moderate steatosis,” and ≥ 60% as “severe steatosis.” In addition, 1-year graft and recipient survival rates were calculated. Graft survival was defined as the absence of recipient death or retransplantation. Early allograft dysfunction was calculated for all recipients and defined as bilirubin ≥ 10mg/dl or INR ≥ 1.6 on POD 7 or AST/ALT >2000 IU/l during the first 7 days [17].

### 2.5. Statistical Analysis

Data is presented as mean ± standard deviation (SD) for normal distribution of data. Not normally distributed data is reported in median and interquartile range. Categorical variables were measured in proportions and counts. After testing for normality, continuous parametric variables were analyzed with the Student's t-test and nonparametric variables using the Wilcoxon rank-sum test. Grouped variables were analyzed with the one-way ANOVA or the Kruskal-Wallis test according to normality. Categorical variables were analyzed using the Pearson *χ*^2^ test.

A binary logistic regression analysis for liver acceptance was carried out. Age was classified into groups of <50 years and above 50 into decades: 50-59, 60-69, 70-79, and >80. BMI was classified in a similar way: < 18.5, 18.5-24.9, 25-29.9, 30-34.9, 35-39.9, and >40. All reported* P* values are two-sided; overall a* P *value < 0.05 was considered significant. Graphs were plotted using GraphPad Prism Version 6.04 for Macintosh (GraphPad Software, La Jolla, CA, USA) and calculations were carried out using IBM SPSS Statistics for Macintosh Version 24.0 (IBM Corp., Armonk, NY, USA).

## 3. Results

### 3.1. Increasing Number of Cancelled Transplantations

**Figure 1:**
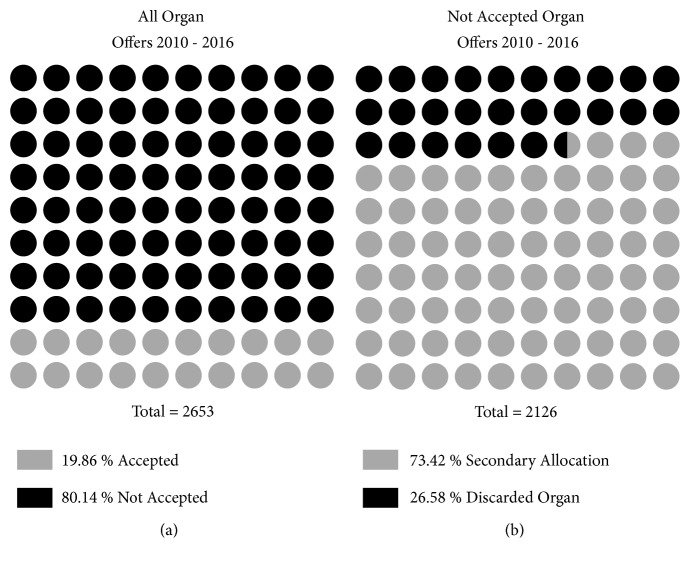
Organs offered and accepted 2010-2016. (a) All liver allografts offered to our center, accepted and not accepted. (b) All nonaccepted liver allografts, secondary allocation to another center or discarded from allocation.

From 2010 to 2016 liver grafts from 2653 donors were offered to the Charité – Universitätsmedizin Berlin. Organs from 527 donors (19.9%) were accepted and successfully transplanted (Figure 1(a)). From the remaining 2126 (80.1%) of offered donor organs, 1561 (73.4%) were allocated and transplanted at other centers and 565 (26.6%) donors could not be allocated and were not used for transplantation (Figure 1(b)). Of all organs not used for transplantation, 304 (53.8%) were not procured at all.

At our center the number of livers accepted and transplanted significantly decreased (p < 0.001) from 2010 (102, i.e., 32.9%) to 2016 (66, i.e., 10.9%), while the number of nonaccepted organ offers more than doubled (208 and 536, respectively) in the same time period. Although the number of patients awaiting OLT at our center decreased from 157 in 2010 to 98 in 2016, respectively, there were more overall offers.

### 3.2. Reasons for Liver Allograft Nonacceptance

Medical issues of the donors, such as age, biochemical parameters, or steatosis hepatis, were the primary reason for graft nonacceptance. Steatosis hepatis, as reported by the explant surgeon based on macroscopic or histopathological assessment of the graft, did not differ significantly from 2010 (15.0%) to 2016 (11.8%), but the number of cancelled liver transplantations due to macrovesicular steatosis hepatis of the graft significantly increased from 2010 to 2016 (p < 0.001) from 15 to 42.

### 3.3. Donors of Discarded Organs Are Older and Present with Higher Rates of Steatosis Hepatis

**Figure 2:**
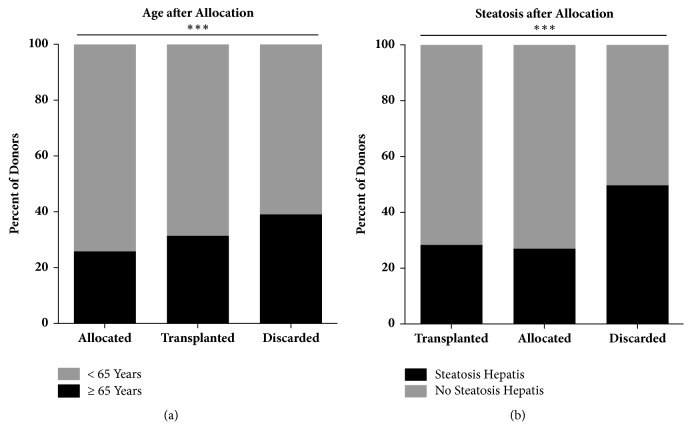
Age and steatosis as influencing factors of acceptance rates. (a) Significantly higher proportion of discarded liver allografts from senior donors (39.1% *∗∗∗* p < 0.001). (b) Steatosis hepatis is significantly more frequently present in discarded organs (49.7%, *∗∗∗* p < 0.001).

In analysis of allocation groups, i.e., transplanted in our center, allocated elsewhere, or discarded, age differed significantly across groups with discarded organs having the highest age (56.0 ± 21.3, p < 0.001). In regard to steatosis hepatis, 49.7% of discarded organs had reports of steatosis hepatis compared to 28.3% transplanted at our center and 27% transplanted elsewhere after secondary allocation (Figure 2(b)). Age and steatosis hepatis were significantly associated (p < 0.001); almost half of donors (45.2%) above the age of 65 had reports of steatosis hepatis, compared to 26.5% in donors under the age of 65 (Figure 2(a)).

Cause of death due to trauma was the least likely cause for organs to be discarded 12.6% vs. 15.9%, 19.8, respectively, p < 0.001). Furthermore, the laboratory parameters AST, GGT, bilirubin, INR, and creatinine were all significantly higher (p < 0.001) in the group of discarded organs (Table 1).

### 3.4. Increasing Prevalence of Steatosis Hepatis in Accepted Organs

**Figure 3:**
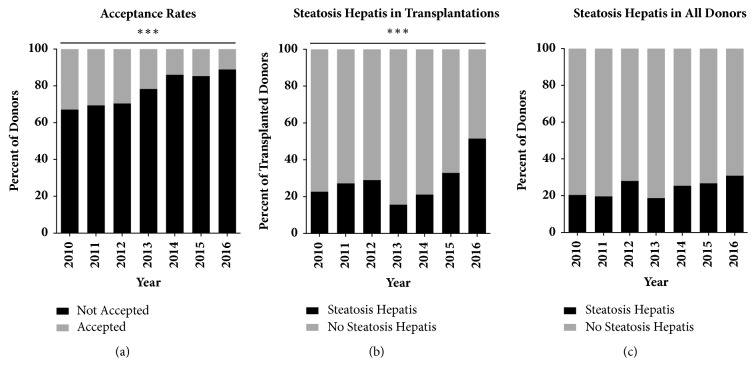
Steatosis hepatis prelavence and influence on acceptance rates. (a) Declining acceptance rate for all liver allografts during study period (*∗∗∗* p < 0.001). (b) Significant increase in steatosis hepatis in transplantations in our center (*∗∗∗* p < 0.001). (c) Trend in rising steatosis hepatis prevalence in donor population after 2012.

From 2010 to 2016 the proportion of accepted and transplanted steatotic livers increased significantly (p < 0.001, Figures 3(a) and 3(b)). While 22.3% (n = 22) of livers transplanted in 2010 had evidence of steatosis hepatis, this number rose to 51.5 % (n = 66) by 2016. During the same time the proportion of discarded organs which were steatotic significantly decreased (p = 0.04) from 61.2% (n = 30) in 2010 to 46.8% (n=66) in 2016. In the overall offered donor pool, steatosis hepatis differed in between years (p = 0.007), with an increase of the prevalence from 24.0% in 2013 to 34.7% in 2016 (Figure 3(c)).

### 3.5. Histopathological Reports of Steatosis Hepatis Influence Acceptance Rates

Histopathological reports of donors were available in 28.9% (n = 766) of cases. In discarded liver grafts 42.5% of organs had a pathology report present compared to only in 24.7% in transplanted liver grafts. Moderate or severe steatosis hepatis was present in discarded organs in 43.8% (n = 105) of cases with 17.5% (n = 42) of those being severely steatotic, i.e., ≥ 60% macrovesicular steatosis (Table 2).

### 3.6. Steatosis Hepatis Is a Significant Predictor of Liver Nonuse in Multivariate Model

A logistic regression was performed to determine the effects of age, BMI, cause of death, history of smoking and/or diabetes, blood levels of AST, GGT, bilirubin, INR, creatinine, HCV, HBV, and steatosis hepatis on the probability of liver grafts being accepted for transplantation. The created model was statistically significant *χ*^2^ = 226.25 p < 0.001 and explained 28.0% (Nagelkerke R^2^) of the variance in organ acceptance while correctly classifying 79.7% of cases (Table 3). Donor age below 50 was a significant predictor of organ acceptance (p = 0.004, OR = 2.77, 95% CI = 1.29-5.53); ages above 50 were not significantly associated with organ acceptance. In cases of normal or overweight donor BMI this was equally the case (normal weight: p = 0.001, OR = 7.32, 95% CI = 2.26-3.3; overweight: p = 0.003, OR = 5.6, 95% CI = 1.77-7.73). Compared to donors with positive HCV antibodies, the odds for acceptance were highest in cases of donors being negative for HCV antibodies (p < 0.001, OR = 11.79, 95% CI = 4.49-30.93)

Additionally, lack of steatosis hepatis increased odds for acceptance significantly (p < 0.001, OR = 1.88, 95% CI = 1.33-2.65). Relevant biochemical parameters were levels of AST, GGT, bilirubin, and INR which all presented increased odds for organ acceptance with lower values.

A similar logistic regression was performed with all cases with available histopathological reports. This model was statistically significant *χ*^2^(4) = 119.19, p < 0.001 and explained 40.2% (Nagelkerke R^2^) of the variance in organ acceptance while correctly classifying 78.8% of cases. Macrovesicular steatosis of the graft below 5% was associated with higher organ acceptance (p < 0.001; OR 16.68, 95% CI = 5.01–55.43). In this model only blood levels of creatinine, AST, bilirubin, HCV status, and age were significantly associated with organ acceptance.

### 3.7. Macrovesicular Steatosis Is Predictive for EAD after Transplantation

**Figure 4:**
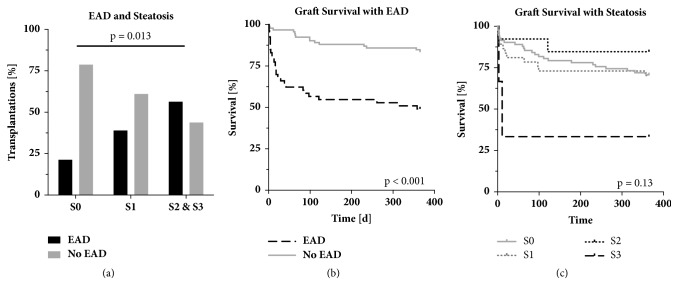
Steatosis hepatis prelavence and influence on graft survival. (a) Increased rates of EAD in steatotic liver grafts (p = 0.013). (b) Kaplan-Meier analysis of graft survival of patients with and without early allograft dysfunction (EAD) (p < 0.001). (c) Kaplan-Meier analysis of graft survival in steatotic liver grafts (p = 0.13). S0, no steatosis; S1, mild steatosis; S2, moderate steatosis; S3, severe steatosis. EAD, early allograft dysfunction.

The incidence of EAD significantly increased (p = 0.013) with regard to the degree of macrovesicular steatosis of the graft. EAD occurred in 13 recipients (21.3%) of grafts with no steatosis, 23 patients (39.0%) that received grafts with mild macrovesicular steatosis, and 9 recipients (56.3%) of grafts with moderate and severe macrovesicular steatosis (Figure 4(a)). Cold ischemia time greater than 8 hours combined with moderate macrovesicular steatosis led to EAD in 9 (69.2%) cases compared to 28 (30.4%) cases with cold ischemia time less than 8 hours (p = 0.006). EAD led to significantly decreased 1-year recipient (p < 0.001) and graft survival (p < 0.001) (Figure 4(b)). In univariate analysis, severe macrovesicular steatosis compared to any other grades of steatosis or no steatosis had a significant effect on 1-year graft survival (p = 0.03; 33.3% vs. 75%) and recipient survival (p = 0.04; 33.3% vs 72.0%), while lower degrees of macrovesicular steatosis had no effect on graft survival (p = 0.13, Figure 4(c)).

## 4. Discussion

The increasing shortage of suitable organs for transplantation necessitates constant reevaluation and expansion of organ acceptance criteria. The results of our study show a steady increase in the overall number of liver graft offers but a decline in the acceptance rate from 2010 to 2016. The increase in the number of organs offered to our center is in huge discrepancy with the factual number of organ donations in the* Eurotransplant* region and the decreasing number of realized liver transplantations. This trend can be explained by multiple or repeating offers of marginal organs from extended criteria donors to different transplant centers and may be attributed to a more restrictive policy for acceptance of organs from extended criteria donors for high-MELD patients, which are prioritized in the* Eurotransplant* allocation system.

Steatosis hepatis is a critical factor for declining an organ offer. The rising prevalence of steatosis hepatis in the general population is well documented [27, 28]: The overall prevalence today is between 20% and 30% in Europe and 46% in the United States [10, 29]. De Graaf et al. reported a rate of 62% microvesicular and 38% macrovesicular steatosis in liver allografts from deceased donors from 2001 to 2007 in Australia [16]. Among potential living donors, the prevalence of biopsy-proven nonalcoholic fatty liver disease ranged from 15% to 53% in different studies and disqualified 3% to 21% of potential liver grafts [10]. In contrast, the extent of steatosis on the nonacceptance rate of donated livers is not well documented in the literature. Of note, such an evaluation is limited by the fact that there is no standardized protocol for evaluation of steatosis hepatis of potential liver grafts. The assessment of liver steatosis in potential deceased donors is usually limited to the sonographic inspection or analysis in computed tomography scans. Histopathological verification cannot be performed on a routine basis in hospitals conducting organ retrieval due to logistic reasons. Even though, in our cohort, if steatosis hepatis was reported from ultrasound or macroscopic examination, it was confirmed in 92.5% of cases with a histopathological analysis, nevertheless, liver grafts are rejected by the transplant center based on a nonstandardized assessment of steatosis hepatis. We here show that even though the proportion of steatotic donor organs transplanted in our recipients increased significantly from 2013 to 2016, 42 liver grafts were still rejected due to steatosis hepatis in 2016, which accounted for a potential increase of 63.6% liver transplantations not transplanted due to steatosis of the graft. Compared to 2010, this rate of not-realized transplantations increased by 49%. Efforts to counteract steatosis hepatis are therefore of immediate clinical relevance and should be therapeutically targeted, e.g., by conditioning of steatotic liver grafts by machine perfusion during the preservation period [30].

Our evaluation of reason for nonacceptance confirms previous reports from Orman et al. concerning age and BMI [9]. However, the history of diabetes mellitus was not significantly associated with organ acceptance in our study cohort. The effect of DCD donors on acceptance could not be investigated, as transplantation in Germany is strictly DBD donors only. Additionally, we could show that a significantly larger proportion of steatotic organs were discarded and that prevalence of steatosis hepatis increased over time. In multivariate analysis, we found a significant trend to declining organs with moderate or severe macrovesicular steatosis and a positive trend for acceptance of organs with macrovesicular steatosis hepatis levels below 30%.

Interestingly enough steatosis hepatis coincided with major limitations in organ quality such as increased liver injury parameters and decreased renal function parameters. Donors with steatosis hepatis also had increased ICU stay duration and were older. This combination is challenging, as each factor for itself is known to negatively impact survival after liver transplantation [15, 31, 32]. Unlike in the United States, recipient survival after liver transplantation has been worse since the introduction of the MELD score [33–35]. Irregularities uncovered in the German liver allocation program since 2012 have changed the donor pool and led to fewer suitable donors and reduced overall organ quality [33]. Our data on graft acceptance rate reflects this trend and shows a 21.8% drop of acceptance from 2010 to 2016, while organ offer numbers doubled in the same time.

Our evaluation of the outcome of macrovesicular liver grafts confirms previous reports, showing significantly higher rates of EAD compared to grafts with no steatosis. Unlike previously reported [16, 23, 36], we observed this effect not only for severe but also for mild and moderate steatotic grafts. However, the definition of EAD is not standardized. As previously reported from Westerkamp et al., the combination of long CIT and moderate or severe macrovesicular steatosis had detrimental effects on the outcome after transplantation [37]. These effects, which we already observe in grafts with mild steatosis, could demand adapted allocation and preservation procedures to reduce CIT and subsequently reduce expected ischemia-reperfusion injury [18, 19].

## 5. Conclusion

In conclusion, our analysis shows a significant decline in liver allograft acceptance rate at our center. In parallel to the increase of steatosis hepatis in the donor pool there was a significant increase of steatosis hepatis acceptance at our center especially in cases with histopathological confirmation of less than 30% macrovesicular steatosis. Moreover, we show a strong association of macrovesicular graft steatosis and the development of EAD, especially in cases with prolonged CIT. Although our results are limited by the retrospective and single center analysis, we propose the present data to be at least relevant for Germany and the* Eurotransplant* region, where high MELD patients and organs from extended criteria donors affect the outcome of liver transplantation. Therapeutic concepts are necessary to address the rising prevalence of steatotic grafts and the inferior outcome associated with transplantation of such grafts. These concepts should range from limiting the effects of cold ischemia to the graft by* ex vivo *machine perfusion to the metabolic reconditioning of steatotic organs prior to transplantation [38].

## Figures and Tables

**Table 1:** Donor data overview.

	**Total**	**Allocated**	**Transplanted**	**Discarded**	***P *value**
N	2653	1561	527	565	
Gender (m) (n, %)	1418 (53.5)	816 (52.3)	268 (51.0)	334 (59.3)	0.007
Age^1^	51.3 ± 20.6	49.0 ± 20.8	53.25 ± 18.2	56.0 ± 21.3	< 0.001
Cause of Death					< 0.001
Trauma (n, %)	464 (17.5)	309 (19.8)	84 (15.9)	71 (12.6)	
Cerebrovascular (n, %)	1308 (49.3)	716 (45.9)	288 (54.6)	304 (53.8)	
Anoxia (n, %)	379 (14.3)	219 (14.0)	74 (14.0)	86 (15.2)	
Other (n, %)	502 (18.9)	317 (20.3)	81 (15.4)	104 (18.4)	
BMI (kg/m2)^1^	25.5 ± 4.9	25.0 ± 4.3	25.6 ± 4.6	26.8 ± 6.3	< 0.001
ICU stay (days)^2^	3.0 (5)	3 (5)	3 (6)	3 (4)	0.25
AST (U/l)^2^	52.0 (76)	49.0 (71)	47.0 (78)	64.5 (102)	< 0.001
ALT (U/l)^2^	34.0 (60)	34.0 (57)	34.0 (59)	36 (70)	0.08
GGT (U/l)^2^	44.0 (93)	39.0 (78)	47.0 (100)	70.0 (161)	< 0.001
Bilirubin (*µ*mol/l)^2^	8.7 (10)	8.2 (10)	9.2 (10)	12.0 (14)	< 0.001
INR^2^	1.18 (02.4)	1.16 (0.25)	1.19 (0.27)	1.2 (0.29)	< 0.001
Creatinine (*µ*mol/l)^2^	70.7 (57.3)	68.0 (55.75)	73.2 (51.85)	79.8 (68.58)	< 0.001
CRP (mg/l)^2^	139.9 (162.65)	136.8 (164.75)	142.5 (152.4)	143.6 (175.23)	0.29
Na+^+^ (mmol/l)^2^	148.0 (76.0)	148.0 (11)	147.0 (10)	148.0 (12)	0.15
CPR (n, %)	177 (11)	128 (8.2)	14 (2.7)	35 (6.2)	< 0.001
Steatosis Hepatis in sonography or pathology (n, %)	828 (32.1)	409 (27.0)	146 (28.3)	273 (49.7)	< 0.001
Steatosis Hepatis confirmed only in pathology (n, %)	665 (26.6)	334 (22.4)	114 (22.9)	217 (42.4)	< 0.001

^1^Data is presented as mean ± standard deviation. ^2^Data is presented as median (interquartile range). Abbreviations: ALT: alanine-aminotransferase; AST: aspartate-aminotransferase; BMI: body mass index; CPR: cardiopulmonary resuscitation; CRP: C-reactive protein; GGT: gamma-glutamyl transferase; ICU: intensive care unit; INR: international normalized ratio; Na+: serum sodium.

**Table 2:** Steatosis hepatis in donors with available histopathological report.

	Organs Discarded	Transplanted	*P* value
n	240	526	
No Steatosis Hepatis (n, %)	42 (17.5%)	183 (34.8%)	< 0.001
Mild Steatosis (n, %)	93 (38.8%)	274 (52.1%)	
Moderate Steatosis (n, %)	63 (26.3%)	50 (9.5%)	
Severe Steatosis (n, %)	42 (17.5%)	19 (3.6%)	

Data is presented as counts (proportions).

**Table 3:** Multivariate logistic regression of organ acceptance.

	Organ Acceptance	
	*P *value	OR (95% CI)
Age (years)		
< 50	0.004	2.77 (1.29; 5.53)
50 – 59	0.372	1.34 (0.68; 2.80)
60 – 69	0.172	1.63 (0.81; 3.30)
70 – 79	0.567	1.22 (0.62; 2.37)
>80	Reference	
BMI		
< 18.5	0.50	1.62 (0.40; 6.52)
18.5 – 24.9	0.001	7.32 (2.26; 23.77)
25.0 – 29.9	0.003	5.60 (1.77; 17.73)
30 – 34.9	0.12	2.56 (0.78; 8.41)
35 – 39.9	0.51	1.61 (0.40; 6.55)
≥ 40.0	Reference	
Cause of Death		
Trauma	0.76	1.72 (0.94; 3.14)
Cerebrovascular Accident	0.72	1.09 (0.68; 1.73)
Anoxia	0.12	1.61 (0.88; 2.94)
Other	Reference	
Smoking (yes)	0.4	1.12 (0.82; 1.67)
Diabetes mellitus (yes)	0.88	0.96 (0.59; 1.57)
No Steatosis hepatis	< 0.001	1.88 (1.33; 2.65)
HCV antibody negative	< 0.001	11.79 (4.49; 30.93)
HBV core antibody negative	0.09	1.62 (0.94; 2.81)
AST	0.01	1.0 (1.0; 1.0)
GGT	< 0.001	1.0 (1.0; 1.0)
Bilirubin	< 0.001	0.97 (0.96; 0.98)
INR	0.04	0.79 (0.62; 0.99)
Creatinine	0.47	1.0 (1.0; 1.0)
Na+	0.07	0.98 (0.96; 1.0)
CRP	0.76	1.0 (1.0; 1.0)

Data is presented as odds ratios (OR) and 95% confidence intervals (CI). Abbreviations: AST: aspartate-aminotransferase; BMI: body mass index; CRP: C-reactive protein; GGT: gamma-glutamyl transferase; HBV: hepatitis B virus; HCV: hepatitis C virus; INR: international normalized ratio; Na+: serum sodium.

## Data Availability

The data used to support the findings of this study are available from the corresponding author upon request.

## Abbreviations

ALT: Alanine-aminotransferase
AST: Aspartate-aminotransferase
BMI: Body mass index
CPR: Cardiopulmonary resuscitation
CRP: C-reactive protein
DBD: Donation after brain death
DCD: Donation after cardiac death
EAD: Early allograft dysfunction
GGT: Gamma-glutamyl transferase
HCV: Hepatitis C virus
HBV: Hepatitis B virus
ICU: Intensive care unit
INR: International normalized ratio
IQR: Interquartile range
OLT: Orthotopic liver transplantation
SD: Standard deviation
UNOS: United Network for Organ Sharing.
